# Supplementary figures and images for: Neuropeptidergic regulation of compulsive ethanol seeking in C. elegans
Source: Sci Rep. 2022 Feb 2;12:1804. doi: 10.1038/s41598-022-05256-1 (PMC8810865; doi:10.1038/s41598-022-05256-1)

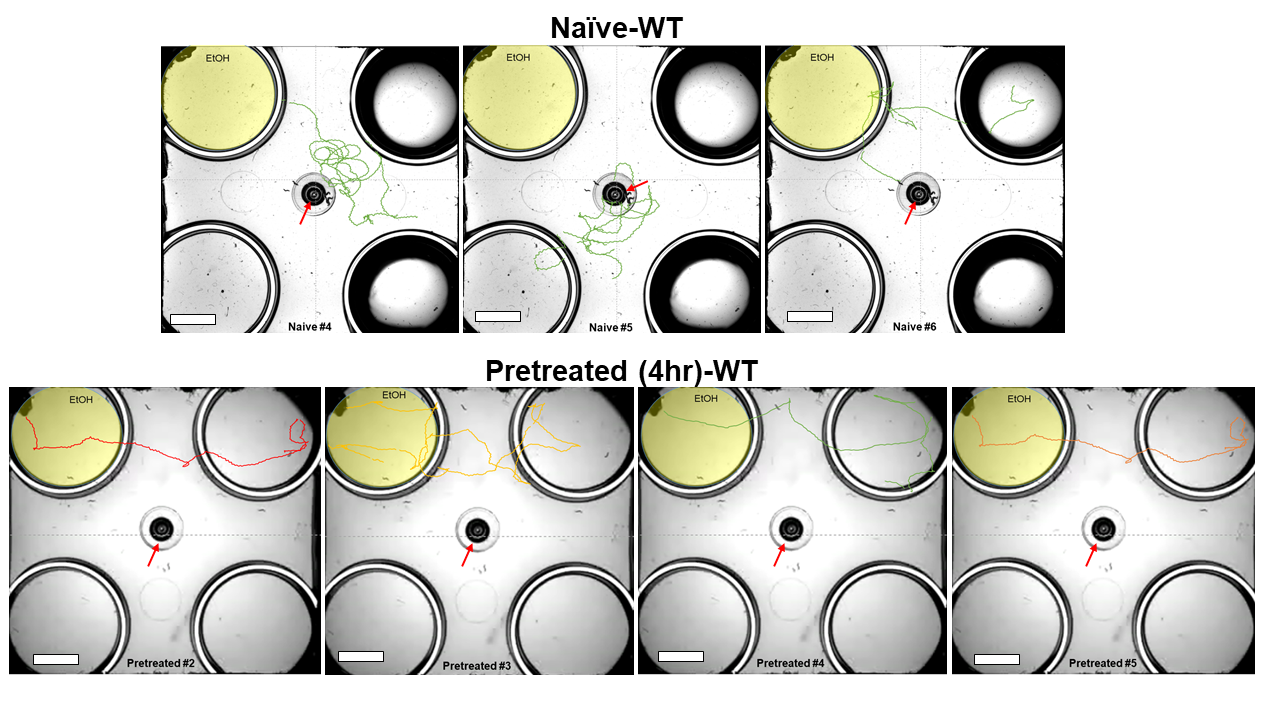

Supplement: Supplementary file 1 — Supplementary Information 1. [file 41598_2022_5256_MOESM1_ESM.tif]

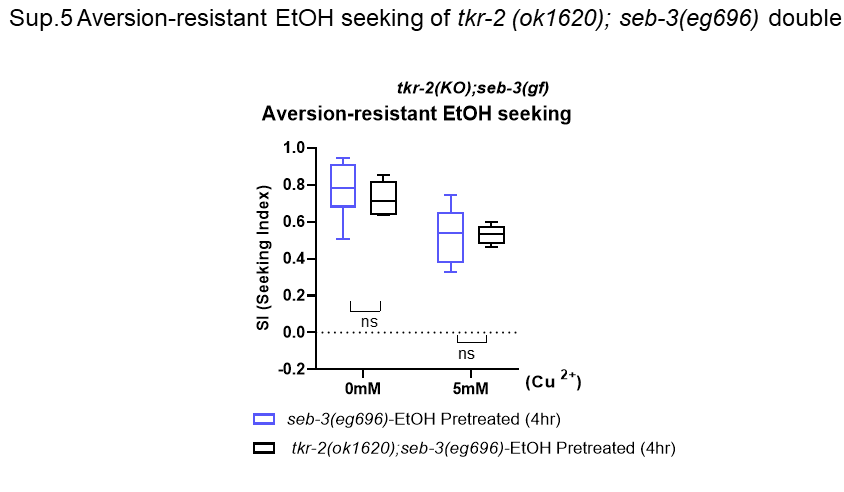

Supplement: Supplementary file 2 — Supplementary Information 2. [file 41598_2022_5256_MOESM2_ESM.tif]

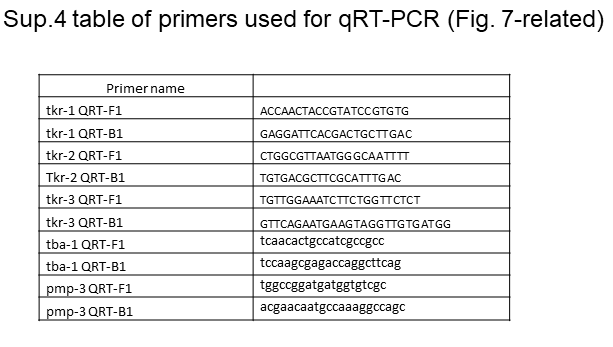

Supplement: Supplementary file 5 — Supplementary Information 5. [file 41598_2022_5256_MOESM5_ESM.tif]
