# Supplementary material for: Neuropeptidergic regulation of compulsive ethanol seeking in C. elegans
Source: Sci Rep. 2022 Feb 2;12:1804. doi: 10.1038/s41598-022-05256-1 (PMC8810865; doi:10.1038/s41598-022-05256-1)
Supplement: Supplementary file 6 — Supplementary Legends. [file 41598_2022_5256_MOESM6_ESM.pdf]

Supplementary Information

Sup. 1. Additional trajectories of individual WT animal (Naïve or Ethanol pretreated). The naïve (a) or ethanol pretreated animals (b), respectively, were placed in the middle of assay plate that contains ethanol (300 mM) only in the left top well.

Sup. 2. List of upregulated genes associated with GO terms in Fig. 6b

Sup. 3. List of downregulated genes associated with GO terms in Fig. 6c

Sup. 4. table of primers used in qRT-PCR (Fig. 7-related)

Sup. 5. Aversion-resistant EtOH seeking of *tkr-2 (ok1620); seb-3(eg696)* double mutant (Fig. 7-related). A two-way ANOVA comparison [$F_{Genotype}(1, 9)=0.1411$, $p<0.0001$; $F_{Concentration}(1, 9)=26.85$, $p=0.0006$; $F_{Genotype\ x\ Concentration}(1, 9)=0.3825$, $p>0.5$]. post hoc differences (Dunnett's test) is not significant.
